# High rate of sensitization to Kathon CG, detected by patch tests in patients with suspected allergic contact dermatitis^☆^^☆☆^

**DOI:** 10.1016/j.abd.2019.09.026

**Published:** 2020-01-31

**Authors:** Eliane Aparecida Silva, Marcia Regina Miras Bosco, Rejane Rojas Lozano, Ana Carla Pereira Latini, Vânia Nieto Brito de Souza

**Affiliations:** a Sector of Immunology, Instituto Lauro de Souza Lima, Bauru, SP, Brazil; b Sector of Pharmacology, Instituto Lauro de Souza Lima, Bauru, SP, Brazil

**Keywords:** Additives in cosmetics, Dermatitis, allergic contact, Hypersensitivity, delayed, Skin tests

## Abstract

**Background:**

Kathon CG, a combination of methylchloroisothiazolinone and methylisothiazolinone, is widely used as preservative in cosmetics, as well in household cleaning products, industrial products such as paints and glues. It has emerged as an important sensitizing agent in allergic contact dermatitis.

**Objectives:**

This study evaluated the reactivity to this substance in patients subjected to patch tests at the Dermatology Institute in Bauru, São Paulo from 2015 to 2017 and its correlation with other preservatives, the professional activity and location of the lesions.

**Methods:**

The patients were submitted to standard series of epicutaneous tests, standardized by the Brazilian Group Studies on Contact Dermatitis.

**Results:**

Out the 267 patients tested, 192 presented positivity to at least one substance and 29 of the patients (15.10%) presented reaction to Kathon CG, with predominance of the female gender (*n* = 27); main professional activity associated with Kathon CG sensibilization was cleaning (17.24%), followed by aesthetic areas (13.79%) and health care (10.34%). The most prevalent sensitizations among the substances tested were nickel sulphate (56.3%), followed by cobalt chloride (23.4%), neomycin (18.2%), potassium dichromate (17.7%), thimerosal (14.5%), formaldehyde (13.2%), paraphenylenediamine (9.3%), and fragrance mix (8.3%).

**Study limitations:**

We do not have data from patients that were submitted to patch test a decade ago in order to confront to current data and establish whether or no sensitization to Kathon CG has increased.

**Conclusion:**

High positivity to Kathon CG corroborates the recent findings in the literature, suggesting more attention to concentration of this substance, used in cosmetics and products for domestic use.

## Introduction

Allergic Contact Dermatitis (ACD) is an inflammatory process mediated by immunological mechanisms that has a significant socioeconomic impact,^1^ since it constitutes an important cause of dermatology appointments and even removal of patients from their activities, with great repercussion on the quality of life and occupational commitment.

The range of clinically relevant allergens, which may be organic and inorganic substances, natural or synthetic, has increased, especially in the more industrialized countries. In addition, factors related to lifestyle also influence the occurrence of ACD. Until 1995, it was estimated that six million chemical substances were present in the environment; three thousand had already been cited as contact sensitizers, and 30 were responsible for 80% of the cases of ACD.^1^, ^2^

The prevalence of ACD by a given antigen depends on its sensitizing potential, as well as the frequency and time of exposure. Thus, the sensitization profile of a given population changes constantly as the presence and exposure to the sensitizers changes over time.^3^

Several studies emphasize the importance of identifying the substance that can trigger ACD.^4^, ^5^, ^6^ In this context, recent studies have shown an increase in the sensitization to the commercially-known substance Kathon CG or Euxil K100, a combination of Methylchloroisothiazolinone (MCI) and Methylisothiazolinone (MI).^7^, ^8^ According to Geier et al., this raise could be due to increased sensitization to the MI component.^9^

The MCI/MI combination, consisting of three parts of MCI and one part of MI, is widely used in Brazil, as preservative in concentrations of 0.0015% (15 ppm) in cosmetics, household cleaning and industrial products such as paints and glues. In the standard test series, the substance is used at the concentration of 0.5% in petrolatum.^4^

Data from the North American Allergic Contact Dermatitis Group from 2009 to 2010 revealed a 2.5% rate of MCI/MI sensitization among 4032 patients tested. The global frequency of sensitized patients remained constant around 2.1% from 1998 to 2009, but increased to 3.9% in 2011.^9^, ^10^, ^11^ In 2014 in Brazil, Scherrer and Rocha, demonstrated an increase in positivity to MCI/MI during the 2009–2012 period, where 11.14% of the patients showed positivity to this substance, contrasting with 3.35% positivity in the period 2006–2009.^7^

As ACD to Kathon CG (MCI/MI) is a relatively common dermatosis in adults at the productive phase of life and studies have shown that the frequency of positivity to this compound has increased, we consider relevant to conduct a retrospective study of the results of the patch test performed in patients attended at the Institute Lauro de Souza Lima Institute, Bauru (SP) in order to evaluate the magnitude of the problem in these patients.

## Methods

A retrospective study was carried out at the Immunology Section of Lauro de Souza Lima Institute, Bauru (SP) based on the results of the epicutaneous tests in patients with the diagnostic hypothesis of ACD or other delayed type hypersensitivity reaction with cutaneous impact, during the period of January 2015 to June 2017. The following parameters were evaluated: sex, age, occupational activity, location of the lesions and frequency of sensitization to the substances tested.

The standard series tested are recommended by the Brazilian Group of Studies on Contact Dermatitis (GBEDC, 1996),^4^ manufactured by FDA-Allergenic/Immunothec (RJ, Brazil), composed of 30 substances (Table 1). In all the cases FINN Chambers (Oy, Finland) type retainers were used, and the readings were done in 48 and 96 h, according to the International Contact Dermatitis Research Group (ICDRG, 1981), in accordance with: (−) negative reaction; dubious reaction; (+) mild reaction, with mild erythema and some papules; (++) moderate reaction, with erythema, papules and some vesicles; (+++) intense reaction, with erythema, papules and confluent vesicles. Irritation reactions were not considered.

**Table 1:** Standard Brazilian series of the contact test recommended by the Brazilian Group of Studies on Contact Dermatitis, 1996.

Substance	Concentration	Substance	Concentration
Anthraquinone	2.0%	Neomycin	20.0%
Balsam of Peru	25.0%	Nitrofurazone	1.0%
Benzocaine	5.0%	Parabens (2)	12.0%
Potasssium dichromate	0.5%	Paraphenylenediamine	1.0%
P-tertiary butylphenol	3.0%	Perfume-mix (3)	8.0%
Carba-mix (1)	3.0%	PPD-mix (4)	0.6%
Cobaltchloride	1.0%	Promethazine	1.0%
Colophony	20%	Propyleneglycol	1.0%
Ethylenodiamine	1.0%	Quaternium	2.0%
Formaldehyde	2.0%	Quinoline-mix (5)	5.0%
Hydroquinone	1.0%	Epoxyresin	1.0%
Irgasan	1.0%	Nickelsulphate	5.0%
Kathon CG	0.5%	Turpemtine	10.0%
Lanolin	20.0%	Thimerosol	0.1%
Mercaptobenzothiazole	1.0%	Thiuram-mix (6)	1.0%

* All substances diluted in petrolatun, with the exception of formaldehyde diluted in water.

(1) Diphenylguanidine; (2) Butyl, ethyl, propyl, methyl paraben, 3.0% each; (3) Eugenol, isoeugenol, cinamic alcohol, cinamicaldehide, geraniol, hidroxicitronellal, alpha-amyl cinamic alcohol, oakmoss absolute, 1.0% each; (4) N-phenyl-n-cyclo-hexyl-p-phenylenediamine, N-iso-N-phenyl-p-phenylenediamine, N-diphenyl-p-phenylenediamine, 0.2% each; (5) Clioquinol, clorquinaldol, 3.0% each; (6) tetramethylthiuramdisulfite tetramethylthiurammonosulfite tetraetiltiuramdisulfite dipentametilenethiurammonosulfite, 0.25% each.

These data were inserted into an Excel® (Microsoft®) file, from which the quantification and descriptive analysis of the results was done. Nonparametric statistical calculations using the chi-square test were used to compare proportions with regard to the studies of the Brazilian Group of Contact Dermatitis^4^ and the one accomplished at Santa Casa de São Paulo during 2006–2011.^12^ A binary logistic regression model was used to assess the association of sensitivity to Kathon CG with sex and occupation. The concomitance of positive reactions to different preservative substances was calculated using Fisher's exact test. The significance level adopted was *p* ≤ 0.05.

The study was approved by the Research Ethics Committee of the Lauro de Souza Lima Institute (number: 2 903 882).

## Results

**Figure 1:**
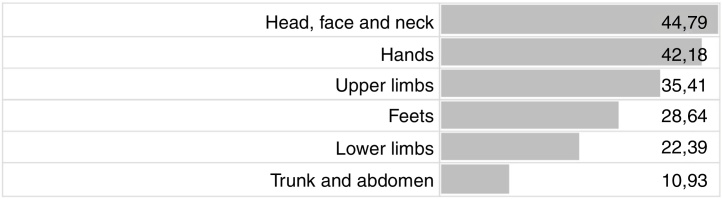
Location and frequency of lesions in patients with positive tests (percentage).

In the evaluated period, 267 patients, with a mean age of 43 ± 16 years of which 191 (72.9%) were female, were submitted to the allergic contact test and, of these, 192 (71.91%) presented positivity to at least one substance tested. Cephalic segment including head, face and neck (44.79%), hands (42.18%) and upper limbs (35.41%) were the most frequently affected location of dermatitis (Fig. 1).

Among the tested substances, the most prevalent was nickel sulphate (56.25%), followed by cobalt chloride (23.43%), neomycin (18.22%), potassium bichromate (17.70%), Kathon CG (15.10%), thimerosal (14.58%), formaldehyde (13.02%), paraphenylenediamine (9.37%) and fragrance mix (8.33%), as shown in table 2.

**Table 2:** Prevalence of reactivity for each sensitizing substance (*n* = 192 patients with positive tests for at least one substance).

Sensitizing substance	Number of positive tests	Prevalence
Nickel sulphate	108	56.25%
Cobalt Chloride	45	23.43%
Neomycin	35	18.22%
Potassium dichromate	34	17.70%
Kathon CG	29	15.10%
Thimerosal	28	14.58%
Formaldehyde	25	13.02%
Paraphenylenediamin	18	9.37%
Fragrance mix	16	8.33%
Colophony	11	5.72%
Balsam of Peru	9	4.68%
Irgasan	8	4.21%
Thiuran mix	6	3.12%
Carba mix	6	3.12%
Ethylenediamine	5	2.60%
Parabens-mix	5	2.60%
Quaternium 15	4	2.08%
Quinoline mix	4	2.08%
Nitrofurazone	4	2.08%
Epoxi resin	4	2.08%
Lanolin	3	1.56%
Mercaptobenzothiazole	3	1.56%
Hydroquinone	2	1.04%
PPD mix	2	1.04%
Promethazine	2	1.04%
Benzocaine	2	1.04%
P-tertiary Buthylphenol	1	0.52%
Turpentine	1	0.52%
Anthraquinone	0	0.0%
Propylene glycol	0	0.0%

When comparing the positivity rates to allergic substances between the groups of patients evaluated in our study and those studied by the Brazilian Group of Contact Dermatitis (2000) and the Santa Casa de São Paulo group (2006–2011), we found a concomitant significant difference of increased positivity for eight substances, with those presenting with the highest positivity being nickel sulfate and cobalt chloride (Table 3).

**Table 3:** Frequency of sensitization with significant difference in relation to two studies in the literature in Brazil (Brazilian Group of Contact Dermatitis^4^ and at Santa Casa of São Paulo during 2006–2011^12^).

Substance significant difference	ILSL study %	GBDC 2000 %	Santa Casa %	Chi-square *p* < 0.05 GBDC × ILSL	Chi-square *p* < 0.05 SC × ILSL
Nickel sulphate	56.25	25.1	28.16	73.4839 (*p* < 0.00001)	50.9494 (*p* < 0.00001)
Cobalt chloride	23.43	11.0	10.52	21.8247 (*p* < 0.000003)	20.8348 (*p* < 0.000005)
Neomycin	18.22	4.3	7.28	49.8009 (*p* < 0.00001)	19.7243 (*p* < 0.000009)
Potassium dichromate	17.7	8.1	11.7	17.0606 (*p* < 0.000004)	5.6508 (*p* < 0.017)
Kathon CG	15.1	2.2	2.43	64.9045 (*p* < 0.00001)	45.8274 (*p* < 0.00001)
Formaldehyde	13.02	3.8	3.24	26.747 (*p* < 0.00001)	26.7292 (*p* < 0.00001)
Colophony	5.72	2.6	2.75	5.2608 (*p* < 0.02)	3.8937 (*p* < 0.04)
Irgasan	4.21	0.7	0.81	14.8632 (*p* < 0.00012)	10.4576 (*p* < 0.001)
Total patients with positive tests	192	967	618		

ILSL, Instituto Lauro de Souza Lima.

**Figure 2:**
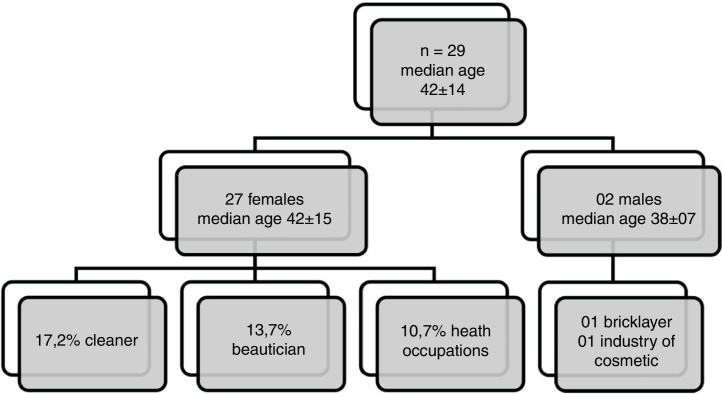
Characteristics of patients with positive results to the Kathon CG (*n* = 29).

Of the 29 patients who presented with a positive reaction to Kathon CG, 93.10% were female (Fig. 2). The main ACD sites of the body of patients with sensitization to Kathon CG were: hands (58.6%), head, face and neck (48.3%), followed by upper limbs (44.8%). The most prevalent professional activity was cleaning (17.24%), followed by beautician (13.79%) and health occupations (10.73%). Among the two male subjects who tested positive for this substance one was a bricklayer and the other worked in the cosmetics industry. By using binary logistic regression we observed that the positivity to Kathon CG is related to females (OR = 9.7; 95% CI: 1.3–76.9); however, no association with occupation was observed in this study.

Regarding the response obtained in reading this test, we identified 65.5% of patients with possible relevance to Kathon CG, because the positivity is associated with the use of the material by the patient.

When we evaluated the reactivity to other preservative substances tested in the standard battery we found positivity of 13.02% to formaldehyde, 4.21% to Irgasan, 2.60% to paraben and 2.08% to quartenium 15. However, with regard to concomitance of these preservatives with Kathon CG, we observed 27.58% of reactivity to formaldehyde among Kathon CG reactive patients, which was statistically significant (*p* = 0.0304) and 6.89% positivity for each of the other preservatives substances.

## Discussion

Kathon CG is currently one of the most frequent causes of contact allergy to preservatives due to its wide and generalized use in both the private and professional settings.^8^, ^9^ In this context, our study verified a high positivity to this substance (15.10%) in the patients tested, which is in agreement with a retrospective study carried out in Brazil that showed 11.14% of sensitization for MCI/MI in the 2009–2012 period, contrasting with (3.35%) for the previous period.^7^ This may be due to the widespread use of this substance in cosmetics and household products in Brazil.

Cosmetics are continuous use products that eventually may trigger hypersensitivity reactions in genetically predisposed individuals, but the allergic reaction cannot be explained only by genetic factors, being considered multifactorial.^13^, ^14^ Thus, the higher occurrence of Kathon CG positivity in females can be related to the sensitization due to frequent use of cosmetics, as observed in the study by Pónyai et al. (2016).^15^

Since the introduction of MCI/MI in the 1980s,^1^, ^16^ this product has manifested itself as a potent sensitizer, which now has a reduced maximum concentration in cosmetics down to 15 ppm.^4^ The frequency of sensitization remained stable in Europe between 1% and 4%^17^ and in Spain between 3% and 4%^18^, ^19^ until 2008. Since then the frequency of sensitization has doubled to reach 8% in 2012 in Spain.^20^ Our data show an even higher sensitization of 15.10%.

The site of eczema is of great importance, with dermatitis on the face being usually related to cosmetics and on hands with occupational factors.^21^ Our study confirms such data in relation to MCI/MI: the hands are the most affected site, followed by the head and neck area, suggesting exposure to agents that cause allergy such as shampoos, creams, cleaning products (detergents, stain removers and fabric softeners). It should be mentioned that MCI/MI is also present in metallurgical fluids, wall paints, lacquers, printer inks and glues,^22^ which could justify the sources of sensitization of the bricklayer patient whose sites affected were arms and hands.

Regarding the age range of patients (mean age 43 ± 16 years), it is consistent with the period of greatest professional activity in the population. This demonstrates the need for greater awareness for the employed population and employer concerning the monitoring of occupational allergies, their risks and effects in the long term.

In the present study, MCI/MI contact allergy was also compared to the reactions of other preservative substances present in the patch test. Our findings reinforce association and show concomitance between MCI/MI and formaldehyde (*p* = 0.0304). The literature has shown an association between contact allergy to formaldehyde and MCI/MI in previous studies.^23^, ^24^ The concomitance of positive tests to these elements occurs, most of the time, by co-sensitization, due to the simultaneous exposure to different materials containing these elements. In this context, our findings reflect consumer behaviour in relation to cosmetics, as well as domestic cleaning products.^25^, ^26^ It is worth mentioning that MCI/MI and formaldehyde are also present in work environments, and the sensitization to these agents can cause occupational contact dermatitis, sometimes due to unexpected exposure, such as the case of one of the male patients whose professional activity is in the building sector, where he could come in contact with water-based paint, lacquer and products for wood polishes.

From the correct identification of the cosmetic components and the industrialized products triggering allergies, the patient should be appropriately oriented in relation to the chemical name of the substance, synonyms and products where their presence occurs and main ways of avoiding exposure.

## Conclusion

Our results show high positivity to Kathon CG and support the recent findings of the literature. Moreover, they suggest greater attention to the concentration of MCI/MI used in cosmetics, as well as in products intended for domestic use.

## Financial support

None declared.

## Authors’ contributions

Eliane Aparecida Silva: Statistic analysis; elaboration and writing of the manuscript; effective participation in research orientation.

Marcia Regina Miras Bosco: Obtaining, analysis, and interpretation of the data; effective participation in research orientation.

Rejane Rojas Lozano: Obtaining, analysis, and interpretation of the data.

Ana Carla Pereira Latini: Statistic analysis; approval of the final version of the manuscript; obtaining, analysis, and interpretation of the data; critical review of the literature.

Vânia Nieto Brito de Souza: Approval of the final version of the manuscript; critical review of the literature; critical review of the manuscript.

## Conflicts of interest

None declared.

## References

[1] De Groot A.V. (1996). Patch Testing: tests concentrations and vehicles for 3500 allergens.

[2] Reischel R., Fisher A.A. (1996). Contact dermatites.

[3] Ayala F., Balato N., Lembo G., Patruno C., Fabbrocini G., Nofroni I. (1996). Statistical evaluation of the persistence of acquired hypersensitivity by standardized patch tests. Contact Dermatitis.

[4] Grupo Brasileiro de Estudo em Dermatite de Contato. Estudo multicêntrico para elaboração de uma bateria padrão brasileira de teste de contato. An Bras Dermatol. 2000;75:147–56.

[5] Duarte I., Proença N.G. (1989). Utilização da bateria de testes epicutâneos preconizados pelo International Contact Dermatitis Research Group (ICDRG) em população não selecionada de São Paulo. An Bras Dermatol.

[6] Serra Baldrich E., Lluch M., Valero A., Malet A., Gimenez Camarasa J.M. (1995). Contact dermatitis: clinical review of 800 patients tested using the standard European series. Allergol Immunopathol (Madr).

[7] Scherrer M.A., Rocha V.B. (2014). Increasing trend of sensitization to Methylchloroisothiazolinone/Methylisothiazolinone (MCI/MI). An Bras Dermatol.

[8] Urwin R., Wilkinson M. (2013). Methylchloroisothiazolinone and methylisothiazolinone contact allergy: a new epidemic. Contact Dermatitis.

[9] Geier J., Lessmann H., Schnuch A., Uter W. (2012). Recent increase in allergic reactions to methylchloroisothiazolinone/methylisothiazolinone: is methylisothiazolinone the culprit?. Contact Dermatitis.

[10] Mowad C.M. (2000). Methylchloroisothiazolinone revisited. Am J Contact Dermat.

[11] Lundov M.D., Thyssen J.P., Zachariae C., Johansen J.D. (2010). Prevalence and cause of methylisothiazolinone contact allergy. Contact Dermatitis.

[12] Duarte I.A., Tanaka G.M., Suzuki N.M., Lazzarini R., Lopes A.S., Volpini B.M. (2013). Patch test standard series recommended by the Brazilian Contact Dermatitis Study Group during the 2006–2011 period. An Bras Dermatol.

[13] Duarte I., Lazzarini R., Buense R., Pires M.C. (2000). Dermatite de contato. An Bras Dermatol.

[14] Schnuch A., Westphal G., Mössner R., Uter W., Reich K. (2011). Genetic factors in contact allergy – review and future goals. Contact Dermatitis.

[15] Pónyai G., Németh I., Temesvári E. (2016). Methylchloroisothiazolinone/methylisothiazolinone and methylisothiazolinone sensitivity in Hungary. Dermatol Res Pract.

[16] Lundov M.D., Krongaard T., Menné T.L., Johansen J.D. (2011). Methylisothiazolinone contact allergy: a review. Br J Dermatol.

[17] Uter W., Aberer W., Armario Hita J.C., Fernandez Vozmediano J.M., Ayala F., Balato A. (2012). Current patch test results with the European baseline series and extensions to it from the “European Surveillance System on Contact Allergy” network, 2007–2008. Contact Dermatitis.

[18] García-Bravo B., Conde-Salazar L., de la Cuadra J., Fernández-Redondo V., Fernández-Vozmediano J.M., Guimaraens D. (2004). Estudio epidemiológico de la dermatitis alérgica de contacto em España (2001). Actas Dermo Sifiliogr.

[19] García Gavín J., Armario Hita J.C., Fernández Redondo V., Fernández Vozmediano J.M., Sánchez Pérez J., Silvestre J.F. (2011). Epidemiología del eczema de contacto en España, Resultados de la Red Española de Vigilancia en Alergia de Contacto (REVAC) durante el año 2008. Actas Dermosifiliogr.

[20] Hervella Garcés M. (2013). Estudio multicéntrico del GEIDAC con la serieestándar de pruebas alérgicas de contacto en 2012. 59 Reunióndel Grupo Español en Investigación de Dermatitis de Contacto y Alergia Cutánea.

[21] Sampaio S.A.P., Rivitti E.A. (2008). Eczema ou Dermatite Eczematosa de Contato. Dermatologia.

[22] Mose A.P., Lundov M.D., Zachariae C., Menne T., Veien N.K., LaurbergG (2012). Occupational contact dermatitis in painters: an analysis of patch test data from the Danish Contact Dermatitis Group. Contact Dermatitis.

[23] Statham B.N., Smith E.V., Bodger O.G., Green C.M., King C.m., Ormerod A.D. (2010). Concomitant contact allergy to methylchloroisothiazolinone/methylisothiazolinone and formaldehyde releasing preservatives. Contact Dermatitis.

[24] Pontén A., Bruze M., Engfeldt M., Hauksson I., Isaksson M. (2016). Concomitant contact allergies to formaldehyde, methylchloroisothiazolinone/methylisothiazolinone, methylisothiazolinone, and fragrance mixes I and II. Contact Dermatitis.

[25] Duarte I., Cunha J., Lazzarini R. (2011). Allergic contact dermatitis in private practice: what are the main sensitizers?. Dermatitis.

[26] Dinkloh A., Worm M., Geier J., Schnuch A., Wollenberg A. (2015). Contact sensitization in patients with suspected cosmetic intolerance: results of the IVDK 2006–2011. J Eur Acad Dermatol Venereol.

